# Coccidioidomycosis Outbreak Among Workers Constructing a Solar Power Farm — Monterey County, California, 2016–2017

**DOI:** 10.15585/mmwr.mm6733a4

**Published:** 2018-08-24

**Authors:** Rebecca L. Laws, Gail Sondermeyer Cooksey, Seema Jain, Jason Wilken, Jennifer McNary, Edward Moreno, Kristy Michie, Christy Mulkerin, Ann McDowell, Duc Vugia, Barbara Materna

**Affiliations:** ^1^Epidemic Intelligence Service, CDC; ^2^California Department of Public Health, Richmond, California; ^3^Office of Public Health Preparedness and Emergency Response, CDC; ^4^Monterey County Health Department, Salinas, California; ^5^San Luis Obispo Public Health Department, San Luis Obispo, California.

## Abstract

In January 2017, two local health departments notified the California Department of Public Health (CDPH) of three cases of coccidioidomycosis among workers constructing a solar power installation (solar farm) in southeastern Monterey County. Coccidioidomycosis, or Valley fever, is an infection caused by inhalation of the soil-dwelling fungus *Coccidioides*, which is endemic in the southwestern United States, including California. After a 1–3 week incubation period, coccidioidomycosis most often causes influenza-like symptoms or pneumonia, but rarely can lead to severe disseminated disease or death (*1*). Persons living, working, or traveling in areas where *Coccidioides* is endemic can inhale fungal spores; workers who are performing soil-disturbing activities are particularly at risk. CDPH previously investigated one outbreak among solar farm construction workers that started in 2011 and made recommendations for reducing risk for infection, including worker education, dust suppression, and use of personal protective equipment (*2*,*3*). For the current outbreak, the CDPH, in collaboration with Monterey County and San Luis Obispo County public health departments, conducted an investigation that identified nine laboratory-confirmed cases of coccidioidomycosis among 2,410 solar farm employees and calculated a worksite-specific incidence rate that was substantially higher than background county rates, suggesting that illness was work-related. The investigation assessed risk factors for potential occupational exposures to identify methods to prevent further workplace illness.

## Investigation and Results

Preconstruction preparations at the approximately 3,000-acre solar farm in Monterey County began in February 2016, and construction started in June 2016 in two phases; the first was completed in August 2017, and the second is expected to continue through the end of 2018. A confirmed case of coccidioidomycosis was defined by the Council of State and Territorial Epidemiologists as a diagnosed illness that met clinical criteria for coccidioidomycosis and was laboratory confirmed (*4*); CDPH further required that illness occurred in a solar farm construction employee, with symptom onset ≥1 week after beginning work and <1 month after the final workday at the solar farm. Employee rosters for February 2016–April 2017 provided by the solar farm owner were matched with the statewide CDPH coccidioidomycosis surveillance database to aid in case-finding. Patients identified through this matching process were interviewed using a structured questionnaire to obtain information on clinical signs and symptoms, occupational exposures, and use of dust control measures at the workplace. Medical records were requested from health care providers, and data were abstracted to confirm that patients met clinical and laboratory criteria. Employee rosters and interviews were used to confirm timing of illness onset associated with solar farm employment. The incidence rate among solar farm workers was calculated by dividing the number of confirmed cases by total person-years spent on the worksite among all employees during the period covered by owner-provided rosters. To calculate person-years, the total number of days between first and last day onsite for each employee was obtained from rosters; total person-days was then converted to person-years by dividing by 365. The annualized incidence among employees at this worksite was compared with background 2016 rates for Monterey and other counties surrounding the worksite by calculating a rate ratio with 95% confidence interval (CI) for each comparison county.

Among 2,410 employees who had worked at the solar farm for ≥1 day, 16 matches between employee rosters and the CDPH coccidioidomycosis surveillance database were identified; medical records were obtained for all 16, and 11 persons were interviewed by telephone. Overall, nine confirmed cases of coccidioidomycosis were identified among the 16 patients; three persons did not meet clinical criteria, and four did not meet work-related illness onset criteria. Eight of nine patients with confirmed coccidioidomycosis were interviewed; one could not be reached after multiple attempts and was confirmed by review of medical records and employment rosters only. Among the nine confirmed cases, median patient age was 42 years (interquartile range = 31–46 years), and seven were male (Table 1). Patients resided in four California counties (Fresno, Madera, Monterey, and San Luis Obispo). Six received diagnoses of coccidioidomycosis pneumonia; five had visited emergency departments from one to four times; one was hospitalized; and none died. Among the eight interviewed patients, seven reported missing work because of illness (median: 14 days; range = 1–320 days).

**TABLE 1:** Demographic and clinical characteristics of patients with confirmed coccidioidomycosis (N = 9) among workers constructing a solar power farm ― California, 2016–2017

Characteristic	Patients, No. (%)
Age (yrs), Median IQR	42 (31–46)
Sex	
Male	7 (78)
Female	2 (22)
Received pneumonia diagnosis	6 (67)
Visited emergency department	5 (56)
Hospitalized	1 (11)
Died	0 (0)

**Abbreviation:** IQR = interquartile range.

Illness onset for the nine patients occurred during August–December 2016 (Figure). Seasonal rains, which suppressed dust, began in late December 2016 and continued through mid-April 2017; most of the first phase of construction was completed by May 2017. All patients reported working outdoors at the solar farm and had job titles that included biologist, paleontologist, electrician, truck driver, iron worker, and general laborer. All eight patients interviewed reported high dust levels frequently (every day or once a week); seven reported that water trucks were frequently unable to control dust levels; five reported frequently working in or near a ditch or trench; no patient was assigned to another work location or sent home because of high dust levels. Seven reported both infrequent (sometimes, rarely, or never) use of respiratory protection and no respirator fit-testing. Although seven reported receiving safety training about Valley fever, only three patients knew what to do if they had symptoms; subsequent review of training materials identified deficiencies (e.g., not emphasizing the potential for Valley fever to be a severe illness, not describing prevention strategies, and not indicating where employees should seek clinical care). No patients reported exposure to a dust cloud or other source of dust or dirt outside of work during the 4 weeks before illness onset.

**FIGURE:**
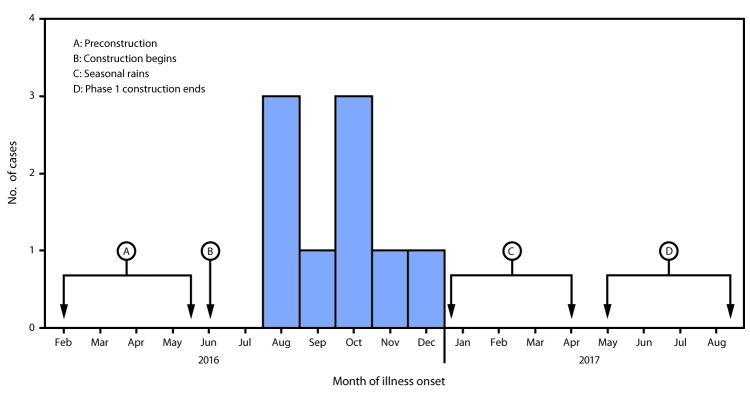
Construction schedule and illness onset of coccidioidomycosis among workers constructing a solar power farm (N = 9) ― Monterey County, California, 2016–2017

The annualized coccidioidomycosis incidence among solar farm workers at this worksite was 1,095 per 100,000 persons/year; whereas the 2016 incidence in Monterey County was 17.5 per 100,000 population, corresponding to a rate ratio of 62.6 (95% CI = 31.4–124.8). Rate ratios for the five counties surrounding the worksite (Fresno, Kern, Kings, San Benito, and San Luis Obispo) ranged from 4.4 to 210.6 (Table 2). These findings indicate that the coccidioidomycosis incidence among employees was significantly higher than the background incidence rates in surrounding counties.

**TABLE 2:** Coccidioidomycosis incidence* and rate ratios among solar farm workers and counties surrounding the solar farm worksite

Population	Incidence (cases per 100,000 population)	RR (95% CI)
Solar farm workers	1,095	—
Monterey County	17.5	62.6 (31.4–124.8)
Kern County	251.7	4.4 (2.3–8.4)
Kings County	157.3	7.0 (3.6–13.5)
San Luis Obispo County	82.8	13.2 (6.8–25.7)
Fresno County	60.8	18.0 (9.3–34.8)
San Benito County	5.2	210.6 (57.0–777.8)

**Abbreviations:** CI = confidence interval; RR = rate ratio.

* Incidence for solar farm workers is annualized. Incidence in surrounding counties is for 2016.

## Public Health Response

On July 26, 2017, CDPH provided interim recommendations for prevention of illness to the solar farm owner and all employers and union representatives associated with the worksite. On August 8, 2017, CDPH and San Luis Obispo Public Health Department conducted a site visit to the solar farm to observe and interview current workers and employers about work practices, dust control, and use of protective equipment; review training materials; and discuss prevention strategies. The visit confirmed dust control issues, serious lapses in use of respiratory protection, insufficient coccidioidomycosis employee training, and no system for tracking or reporting illness. In November 2017, CDPH issued formal investigation findings and prevention recommendations before the start of the second construction phase, which is scheduled to continue through the end of 2018. Recommendations for employers included 1) reducing dust exposure by ensuring ample and efficient water truck capacity to wet soil; 2) using only heavy equipment with enclosed cabs and temperature-controlled, high efficiency particulate air–filtered air; 3) providing clean coveralls daily to employees who disturb soil; 4) implementing a mandatory respiratory protection program (8 CCR ^§^5144, Respiratory Protection: https://www.dir.ca.gov/title8/5144.html) that specifically requires National Institute for Occupational Safety and Health–approved respirators be worn while performing or in the near vicinity of job activities that create airborne dust; 5) developing effective Valley fever training for all employees, including ways to reduce exposure, how to recognize symptoms, and where to seek care; and 6) tracking and reporting of all suspected Valley fever illnesses that occur at the worksite to the Monterey County Health Department. The California Division of Occupational Safety and Health cited six solar farm employers for not protecting workers from coccidioidomycosis; violations included failure to control employee exposure to dust and failure to provide and ensure use of respiratory protection (*5*).

## Discussion

Coccidioidomycosis is a reportable disease in 22 states including California, where a substantial increase in incidence has been observed since 2014 (*6*). Underrecognition, misdiagnosis, and substantial delays between seeking health care and accurate diagnosis are common (*7*). Outdoor workers performing soil-disturbing activities in areas where *Coccidioides* is endemic are particularly at risk for infection, and approximately half of all reported outbreaks have involved occupational exposures (*8*), including outbreaks among workers constructing two solar farms in San Luis Obispo County, California, during 2011–2014 (*2*,*3*).

The high incidence among these solar farm workers provides evidence that coccidioidomycosis was likely acquired at work rather than in the community. Employers in areas with endemic *Coccidioides* should implement infection prevention measures and protect workers who are at risk for exposure to *Coccidioides*; risk for infection can be decreased by using dust-control measures and appropriate personal protective equipment at work (*3*). In California, numerous large-scale energy projects are in the planning or construction phases, and many are in the Central Valley and Central Coast regions where *Coccidioides* is endemic (*9*). Despite previous outbreak investigations and subsequent recommendations (*2*,*3*), this report of another coccidioidomycosis outbreak among solar farm workers indicates that prevention methods need to be better incorporated into the planning and monitoring of construction projects in areas with endemic *Coccidioides* (e.g., by involving public health practitioners in preproject reviews). Outdoor workers in these areas should be trained by employers about the potential for infection, how to limit dust exposure, how to recognize symptoms, where to seek care, and how to ask a health care provider to assess them for coccidioidomycosis. Clinicians should inquire about occupational history and should suspect coccidioidomycosis in patients who are outdoor workers in areas with endemic *Coccidioides* and who have a clinically compatible illness.

**Summary**

**What is already known about this topic?**

Workers performing soil-disturbing activities are at risk for coccidioidomycosis, an infection caused by inhaling the soil-dwelling fungus *Coccidioides*.

**What is added by this report?**

Nine confirmed coccidioidomycosis cases were identified among 2,410 solar farm workers in California. The incidence among workers (1,095 per 100,000 persons/year) was 4.4 to 210.6 times higher than background county rates, providing evidence that illness was work-related.

**What are the implications for public health practice?**

Employers should take measures to protect workers from dust exposure in areas where *Coccidioides* is endemic, involvement of public health practitioners is needed in the review of proposed construction that might expose workers to coccidioidomycosis, and clinicians should suspect coccidioidomycosis in patients with a clinically compatible illness who work outdoors.
